# Respiratory Muscle Function and Exercise Performance in Adult Patients with Fontan Circulation

**DOI:** 10.3390/jcm12144593

**Published:** 2023-07-10

**Authors:** Raquel Ladrón-Abia, Pilar Cejudo Ramos, Israel Valverde Pérez, Rocío Camacho Fernández De Liger, Amadeo-José Wals-Rodríguez, María José Rodríguez Puras, Begoña Manso García, Pastora Gallego

**Affiliations:** 1 Adult Congenital Heart Disease Unit, Hospital Universitario Virgen del Rocío, Instituto de BioMedicina de Sevilla (IBIS), Avenida Manuel Siurot s/n, 41013 Sevilla, Spain; 2 Medical-Surgical Service of Respiratory Diseases, Hospital Universitario Virgen del Rocío, Instituto de BioMedicina de Sevilla (IBIS), 41013 Sevilla, Spain; 3 Paediatric Cardiology Unit, Hospital Infantil Virgen del Rocío, Instituto de BioMedicina de Sevilla (IBIS), 41013 Sevilla, Spain

**Keywords:** adult Fontan patients, exercise capacity, oxygen uptake efficiency slope, lung function, inspiratory muscle weakness

## Abstract

At rest, a good Fontan circulation can provide a normal cardiac output (CO). However, as a consequence of its unique hemodynamic nature, the limitations of the Fontan circuit are exposed during exercise. We aimed to provide a comprehensive assessment of the pathophysiology of exercise in adult Fontan patients (FPs) and identify factors limiting their functional capacity (FC). In a single-center study conducted in 37 FPs aged ≥16 years and 19 healthy-controls (HCs) who underwent CPET on a cycle ergometer in February and March 2022, the mean peakVO_2_ was 21 ± 5.4 mL/kg/min, which was 55% of the predicted value. Morphologically, the left single ventricle showed a higher peakVO_2_% predicted value (57.4 ± 14.4% vs. 43.4 ± 8.1%, *p* = 0.045). The factors associated with low peakVO_2_ values were an early flattened or descending O_2_ pulse at maximal exertion (52 ± 14% vs. 62 ± 12.5, *p* = 0.04 and 47.6 ± 9% vs. 60 ± 14, *p* = 0.018, respectively) and chronotropic insufficiency (53 ± 12% vs. 69.8 ± 20%, *p* = 0.008). The OUES was found to be a useful parameter to assess the FC in FPs in maximal and submaximal exercise testing. A strong positive correlation was observed between the %OUES and peakVO_2_%predicted (r = 0.726, *p* > 0.001). The lung function was impaired in the FPs, mostly with a mild restrictive pattern (56.8%). The FPs showed lower inspiratory muscle strength compared to the HCs but it was not statistically associated with either the peakVO_2_ or VE/VCO_2_ slope. Regular intense physical activity improves one’s FC. Although FPs have inspiratory muscle weakness, its impact on their FC is unclear. The peakVO_2_% predicted grew progressively higher as the level of physical activity increased (low level 49.5 ± 14%, moderate level 55 ± 12%, intense level 69 ± 20%).

## 1. Introduction

Since the introduction of the Fontan operation 50 years ago, the life expectancy of infants born with complex and functionally univentricular congenital heart disease (CHD) has increased significantly, and most children will survive to adult life [1]. The Fontan palliation separates the systemic and pulmonary circulations and relieves cyanosis, with the systemic venous return being connected to the pulmonary arteries without the interposition of the right ventricle pump. At rest, a good Fontan circulation can provide a normal cardiac output (CO). However, as a consequence of its unique hemodynamic, the limitations of the Fontan circuit are exposed during exercise. In these Fontan patients, there is no pump to increase and accelerate the pulmonary blood flow. Furthermore, the pulmonary vascular reactivity and recruitment of vessels are limited or even absent [2]. Additionally, maldistribution of the pulmonary blood flow has been associated with a lower exercise capacity [3]. A patient with Fontan circulation has, therefore, a restricted ability to augment their cardiac output during exercise [4].

Many other factors might contribute to the impaired exercise capacity in patients with Fontan circulation. Thus, in adults with CHD, the lung volumes are commonly reduced, and the forced vital capacity (FVC) correlates with exercise capacity [5,6,7,8] and predicts survival [9]. Moreover, children and young adults with Fontan physiology have impaired growth and pubertal development as well as alterations in body composition, with marked deficits in leg lean mass. A relationship between leg lean mass deficits and exercise capacity has been also described [10,11]. Furthermore, inspiratory muscle training has been an area of recent interest given the importance of inspiration to promote passive flow through the Fontan circulation at rest [12]. Unfortunately, no clear benefit of respiratory muscle training on Fontan circulation has been demonstrated so far, and the research to date has been mixed [13,14,15,16].

Cardiopulmonary exercise testing (CPET) is a valuable tool for assessing the exercise capacity and fitness of individuals with Fontan circulation. It involves measurements of oxygen uptake (VO_2_), carbon dioxide production (VCO_2_), and ventilation during a symptom-limited exercise test. CPET provides clinicians and researchers in an integrative and comprehensive assessment of the physiological responses to exercise and cardiorespiratory fitness in this patient population [17]. The peakVO_2_ measured by CPET is broadly used to classify functional capacity. In different studies, this parameter is strongly related to the outcome and prognosis [18,19,20]. However, it requires a maximal effort for its interpretation, which is not always achieved by adults with Fontan circulation. The oxygen uptake efficiency slope (OUES) is a submaximal parameter that objectively predicts the maximal exercise capacity in both the healthy population and patients with acquired heart disease. The OUES is an effort-independent parameter that is easier to determine than the anaerobic threshold, and unlike peakVO_2_, it does not rely on the last segment of exercise. However, it has only been used in a small number of studies with young Fontan patients [21,22].

Since the OUES uses all of the exercise data, even if submaximal, we sought to study its accuracy as a surrogate marker of peakVO_2_ in maximal and submaximal exercise testing and aimed to provide a comprehensive assessment of the exercise physiology impairments of adults with Fontan circulation using CPET. Furthermore, we evaluated the impact of their physical activity level on their exercise capacity and investigated the contribution of inspiratory muscle weakness to exercise limitations in this cohort of Fontan patients.

## 2. Methods

### 2.1. Data Source and Study Population

All patients aged >16 years who had undergone Fontan palliation and were being followed up at the Adult Congenital Heart Disease Unit in the University Hospital Virgen del Rocio in Seville were identified. Their demographic data, cardiac anatomy, prior therapeutic interventions, complications, diagnostic techniques, and medical treatments were collected from electronic health records and served as the primary data source for this study. Patients with both atriopulmonary and total cavopulmonary connection Fontan circulations, either fenestrated and non-fenestrated, were included. The exclusion criteria were: (a) clinical instability (NYHA functional class IV, protein-losing enteropathy, severe hypoxemia with O_2_ saturation < 80%); (b) arrhythmias in the preceding 6 months prior to inclusion; (c) unstable angina; (d) recent surgery (<12 months) or changes in medication (<6 months), neurological sequelae, cognitive disability, or musculoskeletal problems that prevented us from performing the exercise testing. The presence of valve dysfunction was not considered as an exclusion criterion.

In this cross-sectional study conducted in a single referral center, lung function, inspiratory muscle strength, and cardiopulmonary exercise tests were performed on 37 patients with Fontan circulation who were clinically stable and 19 healthy controls (HCs). The HC participants were non-smokers, were not under drug treatment, and did not have a history of cardiovascular or pulmonary disease. All participants were informed about the details of the procedures, including the potential risks, before signing the written informed consent. The local Ethics Committee on Human Research approved the study.

### 2.2. International Physical Activity Questionnaire

All patients and controls completed the International Physical Activity Questionnaire—Short Form. The IPAQ-SF is a well-developed 7-question instrument that addresses the number of days and time spent on physical activity of moderate intensity, vigorous intensity, and walking for at least 10 min duration over the last 7 days, and includes the time spent sitting on weekdays over the last 7 days [23].

### 2.3. Lung Function

The forced vital capacity (FVC), forced expiratory volume in one second (FEV1), and FEV1/FVC ratio were assessed according to the recommendations of the American Thoracic Society and European Respiratory Society [24] and the predicted values calculated from the equations reported by the Global Lung Function Initiative (GLI) [25]. At least three acceptable and reproducible maneuvers were achieved, using encouragement and positive reinforcement in order to obtain maximum values. The patients with restrictive patterns were classified into 3 groups based on their predicted FVC values, namely mild restriction (predicted FVC 80% to 65%), moderate restriction (FVC 64% to 50%), and severe restriction (FVC < 49%), based on the published recommendations.

### 2.4. Inspiratory Muscle Strength

Maximal static inspiratory pressure (MIP): During testing, the participants were sitting upright. Both the patients and controls were instructed to exhale slowly and completely, seal their lips firmly around the mouthpiece, and then inhale through the mouth as hard and fast as possible, with the nostrils occluded with a clamp. Ideally, the inspiratory pressure was held for 1.5 s so that the maximum pressure sustained for 1 s was recorded. The maximum value of three inspiratory maneuvers that varied by less than 10% were recorded [26]. The reference values were calculated based on the equations developed by Evans et al. [27]: men = 120 − (0.41 × age); women = 108 − (0.61 × age).

Maximal sniff nasal inspiratory pressure (SNIP): The inspiratory pressure was recorded by a pressure transducer connected to a catheter inserted into the nostril. This maneuver consists of sniffing quickly and deeply, generally from the functional residual capacity, then measuring the pressure generated. The duration of the sniff should be <500 ms. The maneuver was repeated 10 times, taking the highest value reached [26]. Reference values in adults between 20 and 80 years of age were calculated from the equations developed by Uldry and Fitting [28]: men = −0.42 × age +126.8; women = −0.22 × age + 94.9. The patients aged 16 and 17 years were calculated with the equations of Stefanutti et al. [29]: men = 3.3 × age + 70.

### 2.5. Cardiopulmonary Exercise Test

Maximal exercise testing was performed using a standardized ramp protocol on an electronically braked cycle ergometer. The participants were submitted to individualized ramp protocols with increments of 5 or 10 watts per minute in Fontan patients and 15 or 20 W/min in healthy controls.

The participants pedaled in an unloaded state for three minutes and the workload was then increased continuously with a slope chosen to achieve each participant’s predicted maximal work rate after 10 to 12 min of cycling. Metabolic measurements were assessed on a breath-by-breath basis throughout the exercise (Ergostik—Geratherm Respiratory GmbH, Bad Kissingen, Germany). A 12-lead electrocardiogram was recorded throughout the exercise test. The blood pressure was measured at rest, every 2 min during exercise, and every minute throughout recovery.

The maximal effort was defined as achieving a respiratory exchange ratio (RER) equal to or greater than 1.10. The CPET was terminated by the patient in cases of discomfort or by the supervising physician in cases of ECG changes, an excessive breathing pattern, or for other reasons. However, the maximal exercise test parameters were calculated only in patients who achieved RER > 1.1. The peak oxygen uptake (peakVO_2_) was determined by the mean of the last 20–30 s of maximal effort. The predicted maximal VO_2_ was calculated by using the Hansen–Wasserman equation [30]. The anaerobic threshold (AT) was determined by the V-slope method, at the point where the linear relationship between CO_2_ production (VCO_2_) and O_2_ consumption (VO_2_) disappears. The VE/VCO_2_ slope was determined until the onset of the respiratory compensation point. The predicted maximum heart rate (HR) was obtained from the 220-age difference. The chronotropic index was calculated by maximal HR-rest HR/(220-age)-rest HR. The oxygen pulse is the VO_2_/HR ratio. The oxygen uptake efficiency slope (OUES) is the slope between the VO_2_ and the logarithmic transformation of ventilation: (VO_2_/log10 VE) − k. The predicted OUES values were obtained using the equation used by Buys et al. [31] developed for Caucasian adults aged 20–60 years: OUESp males = 1093 − 18.5 × age + 1479 × body surface area; OUESp females = 842 − 18.5 × age + 1280 × body surface area. In patients aged 16–20 years, the equation used by Akkerman et al. [32] was used.

### 2.6. Statistical Analysis

Continuous variables are presented as means ± SDs or medians and interquartile ranges in normally and non-normally distributed variables, respectively. Categorical variables are presented as counts and percentages. Comparisons among groups were performed using an unpaired *t*-test or the Mann–Whitney U test in normally and non-normally distributed variables, respectively. An intra-group comparison was performed by applying the *t*-test for paired samples for normally distributed variables or non-parametric Wilcoxon tests for paired samples for non-normally distributed variables. Both Pearson’s correlation coefficient and Spearman’s Rho correlation coefficient were used to measure the correlations between continuous variables. The results were represented by the coefficient of correlation^®^. Statistical significance was considered for *p*-values < 0.05. The data analyses were performed with IBM SPSS 28 statistical software.

## 3. Results

A total of 37 Fontan patients (68% male) with a mean age of 26.5 ± 6.25 years (range 16–41 years) and 19 healthy controls (68.5% men) with a mean age of 26.1 ± 6.8 years were recruited. There were no differences in age, gender, or body mass index (BMI) between the groups. The baseline characteristics of the Fontan patients are displayed in Table 1. The single ventricle morphology was found in 81% of patients. Tricuspid atresia (44%) followed by double inlet left ventricle (27.8%) were the most frequent underlying heart defects. The median age at the time of the Fontan surgery was 8.2 (6.1–11) years. Atrioventricular (AV) valve regurgitation was absent in 14 patients (37.8%), mild in 16 FPs (43%), moderate in 6 FPs (16%), and severe in 1 FP (2.7%). Among the FPs with a single left ventricle and balanced ventricles (32 patients), 62.5% had a preserved ejection fraction (EF), 31.3% had mild systolic dysfunction, and 6.3% had moderate systolic dysfunction. Among the FPs with a single right ventricle, 60% (3 FP) had a preserved EF and 2 FPs (40%) showed a mildly reduced EF. No patient in our cohort had severe systolic dysfunction.

### 3.1. Lung Function

The FVC and FEV1 were significantly lower in Fontan patients compared to their healthy peers. No differences were observed in the FEV1/FVC ratio between both groups. Twenty-one Fontan patients (56.8%) showed a mildly restrictive pattern, six (16.2%) showed a moderate restrictive pattern, and ten (27%) had normal lung function. None of the patients had a severe restrictive or obstructive pattern (Table 2).

The patients with a history of lateral thoracotomy showed a lower FVC (FVC 72 ± 9.5% vs. 80 ± 13%, *p* = 0.047). We did not find a statistically significant association between a reduced FVC and lower peakVO_2_ (*p* = 0.598) or ventilatory efficiency parameters (*p* = 0.545).

### 3.2. Cardiopulmonary Exercise Test

The average time between the Fontan surgery and cardiopulmonary exercise test was 17 ± 5.7 years. While all healthy controls reached their maximal exercise effort (RER ≥ 1.10), up to 8% of the Fontan patients did not achieve a maximal exercise effort (RER < 1.10). Table 3 shows the CPET results for the two groups.

The mean peakVO_2_ was 21 ± 5.4 mL/kg/min, an average of 55% of the predicted peakVO_2_ (peakVO_2_% predicted). The anaerobic threshold (AT) occurred at 32 ± 8% of the predicted peakVO_2_ (early AT). Although there were no differences between healthy subjects and Fontan patients with regard to age, weight, and height, an examination of the peakVO_2_ results revealed 36.6% lower values for the patients than controls, with a mean peakVO_2_% predicted difference of 32 ± 3.8 (*p* < 0.001). The patients with a morphologically left single ventricle showed higher peakVO_2_% predicted values compared to those with a morphologically right ventricle (57.4 ± 14.4% vs. 43.4 ± 8.1%, *p* = 0.045). We found no statistically significant differences in either peak oxygen uptake or VO_2_ at the anaerobic threshold between the different degrees of AV valve insufficiency.

The O_2_ pulse was low, with a mean of 65.7 ± 13.8% of the predicted O_2_ pulse. As for the oxygen pulse kinetics, 51.4% showed an ascending slope and 48.6% showed early flattening after reaching the AT. Furthermore, in 24.3% of the total cohort (9 patients), we observed a descending O_2_ pulse slope at maximal exertion. The patients with an early flattened O_2_ pulse or a descending pulse at maximal exertion had a lower peakVO_2_% predicted values (52 ± 14% vs. 62 ± 12.5, *p* = 0.04; 47.6 ± 9% vs. 60 ± 14, *p* = 0.018, respectively).

The chronotropic response was lower compared to healthy controls, as shown in Table 3. The patients with chronotropic insufficiency had lower peakVO_2_% predicted values (53 ± 12% vs. 69.8 ± 20%, *p* = 0.008). The chronotropic index (r = 0.546, *p* < 0.001), % of maximum predicted HR (r = 0.572, *p* < 0.001), and HR reserve (r = 0.452, *p* = 0.005) showed statistically significant correlations with the peakVO_2_% predicted values. Finally, the Fontan patients had higher values for ventilatory efficiency parameters compared to their healthy peers (Table 3).

### 3.3. Oxygen Uptake Efficiency Slope (OUES)

The mean OUES was 1600 ± 351, a mean of 54% of the predicted OUES (%OUES). A moderate positive correlation was observed between the absolute OUES and absolute peakVO_2_ (r = 0.63, *p* = 0.000). A strong positive correlation was observed between the %OUES and peakVO_2_% predicted values (r = 0.726, *p* > 0.001) and between the indexed OUES/kg and indexed peakVO_2_ (mL/kg/min) (r = 0.846, *p* < 0.001). The OUES also correlated with submaximal parameters: OUES-VO_2_ at the anaerobic threshold (AT) r = 0.608, *p* = 0.000. A negative correlation was seen between the OUES and VE/VCO_2_ slope (r = −0.40, *p* = 0.013).

### 3.4. Physical Activity Level

In our Fontan cohort, 59.5% of the patients had a moderate level of physical activity determined by the IPAQ questionnaire (*n* = 22), while 27% of the cohort (*n* = 10) had a sedentary life and only 13.5% (5 patients) had a high level of physical activity. Among the healthy controls, the physical activity levels according to the IPAQ questionnaire were as follows: low in 7 subjects (37%), moderate in 8 subjects (42%), and high in 4 subjects (21%). No differences were observed between the groups (Table 3).

**Figure 1:**
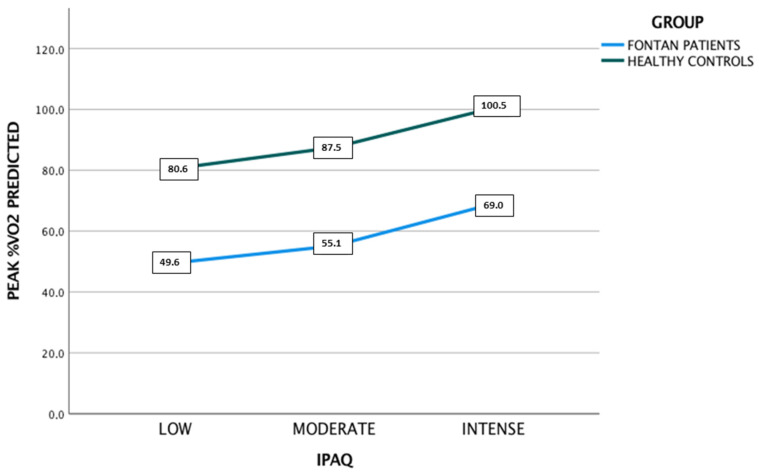
Impact of the level of physical activity on the exercise capacity in Fontan patients and healthy controls. The peakVO_2_% predicted was progressively higher as the level of physical activity increased in both groups. Differences were statistically significant only between low and intense physical activity levels.

The peakVO_2_% predicted values grew progressively higher as the level of physical activity increased (a mean peakVO_2_ at a low activity level of 49.5 ± 14%, at a moderate activity level of 55 ± 12%, at an intense activity level of 69 ± 20%). Although there were differences between the three levels of physical activity, the sample size only allowed us to obtain statistically significant differences between low- and high-intensity activity (mean difference of peakVO_2_% predicted: low-intense physical activity = 19.4 ± 7.5% (*p* = 0.038), low-moderate physical activity = 5.5 ± 5.2%, (*p* = 0.545), and moderate-intense = 13.8 ± 6.8% (*p* = 0.121)). We observed the same pattern of increasing peak oxygen uptake within the different physical activity levels in both the FPs and HCs, as displayed in Figure 1.

Two patients from the total cohort showed a peakVO_2_ greater than 80% of the predicted value, which has been previously dubbed as the “super Fontan”, with the following characteristics: VO_2_ at AT = 49.8 ± 3%, %OUES = 77.8 ± 7%, O_2_ pulse = 91.5 ± 0.7%, peak predicted load = 120 ± 4%, %FCMP = 98 ± 1.2%, chronotropic index = 0.9 ± 0.04, with statistically significant differences from the rest of the cohort. Both patients had a morphologically left single ventricle and performed regular intense physical activity.

### 3.5. Inspiratory Muscle Strength

The SNIP and MIP characteristics are displayed in Table 2. The patients with Fontan circulation showed statistically significantly lower inspiratory muscle strength compared to the healthy subjects. We did not find a statistically significant association between the inspiratory muscle strength and peakVO_2_ or VO_2_ at AT. No significant correlation was observed between the MIP (*p* = 0.482) or SNIP (*p* = 0.570) and VE/VCO_2_ slope or lung function parameters.

## 4. Discussion

In this study, we provide insights into the exercise physiology response, aerobic functional capacity, and factors limiting physical effort in adult patients with Fontan circulation using CPET. Importantly, we demonstrate that the OUES is a submaximal parameter that can be used in Fontan patients, whether or not they achieve a maximal exercise effort. This cohort of patients may also present abnormal lung volumes and inspiratory muscle weakness. Lastly, we demonstrate a correlation between physical activity and exercise capacity in Fontan patients.

### 4.1. Cardiovascular Exercise Performance

#### 4.1.1. Oxygen Uptake

To maintain cardiac output (CO), the Fontan circulation relies on venous pressure to passively drive systemic venous blood through the pulmonary vascular bed to the pulmonary venous atrium, given the absence of a subpulmonary ventricular pump. This intrinsically limits the ability to boost systemic ventricular preload, which leads to a very high prevalence of impaired maximal aerobic exercise capacity [4,33,34]. Along with this, a severe peripheral limitation associated with muscle mass deficit and generalized muscle weakness contribute to exercise intolerance [10,11]. The exercise capacity can be quantitated clinically by measuring the oxygen uptake. Thus, the peakVO_2_ is an established and reliable measure widely used to assess the exercise intolerance of patients with CHD. As our group has previously published [35], the peakVO_2_ is higher on a treadmill than on a cycle ergometer in patients with Fontan circulation (up to 23.8% higher on the treadmill), and this difference in exercise modality should also be taken into account in the assessment of the functional capacity of this population group. Since exercise on a cycle ergometer is less prone to induce noise artifacts in ECG and blood pressure monitoring, with better quantification of the load achieved and more accurate calculation of the parameters derived from it, and as it is less intimidating for people unaccustomed with a moving treadmill, in this study we chose a cycle ergometer as the testing modality. Consistent with previous studies [18,19,20,21,36], we found a significantly worse functional capacity in our population of adults with Fontan circulation compared to the healthy controls, with a mean peakVO_2_ of 21 mL/kg/min, which on average was 55% of what would be predicted for the general population according to the Hansen–Wasserman equation. Importantly, the distribution of the levels of physical activity was similar in the Fontan patients and controls. Given the significance of the differences between CPET variables in Fontan patients and healthy controls, this needs to be highlighted, especially since inactivity in the general population means that many healthy controls might not perform at the predicted levels either. Furthermore, while a reduced peakVO_2_ is commonly observed, there is a subset of Fontan patients who exhibit normal or even supranormal peakVO_2_. Lastly, our study shows that the Fontan patients with a morphologically left single ventricle performed better under CPET. Although it could be hypothesized that the right ventricle is not intended to withstand the physiological pressure and workload required of the systemic ventricle, and conflicting evidence exists on the prognostic significance of the SRV versus SLV after a Fontan operation, a recently published single-center retrospective study suggested that a dominant single ventricle morphology may not be associated with an appreciable difference in exercise performance in adult survivors with a Fontan palliation [37,38]. The small sample size and predominance of FPs with a dominant left ventricle in our study cohort preclude robust conclusions in this respect.

#### 4.1.2. OUES Accuracy

Our cross-sectional study reinforces the notion that several CPET parameters (peakVO_2_, O_2_ pulse, chronotropic incompetence) are helpful in the evaluation of Fontan patients. However, one of its major strengths is that it focuses on the OUES as a surrogate marker for the peakVO_2_ obtained at an RER of 1.1.

When the minute ventilation (VE) over the entire exercise duration is logarithmically transformed and plotted against the VO_2_, the regression coefficient is the oxygen uptake efficiency slope (OUES). This makes the OUES a dimensionless and submaximal parameter of the functional capacity. In addition, the OUES can also be normalized by body weight or body surface area to correct for differences in anthropometrics between patients. In our Fontan cohort, the mean OUES was 1600 ± 351, about 54% of the predicted OUES. Although higher %OUES results of up to 79% of the predicted values have been reported in a pediatric age group [22], the mean %OUES in our study was similar to that reported in older Fontan patients [21]. The strongest correlations were seen between the %OUES and peakVO_2_% predicted and between the OUES/kg and indexed peakVO_2_ (mL/kg/min), as previously demonstrated by Terol et al. in a pediatric Fontan cohort at a median age of 11 years [22], although we also observed a good correlation between the OUES and other parameters of submaximal functional capacity (VO_2_ at AT). Typically, clinical trials and observational studies often use the peakVO_2_ as the primary outcome measure. However, some investigators have proposed using submaximal parameters such as the VO_2_ at AT in Fontan patients as outcome measures instead. Submaximal exertion may be more representative of daily activities, and by the time they have reached peak exercise, the central venous pressure in Fontan patients has hit a physiological limit and may not be amenable to improvement. Moreover, in Fontan patients who are unable to perform a maximal effort, a lower VO_2_ at anaerobic threshold (AT) has been associated with a greater risk of death or transplant, and one study identified a cutoff value of <9 mL/kg/min as being associated with increased mortality. Although in other studies higher percentages of submaximal effort have been described (up to 20% do not reach the maximum effort) [22], in our series 8% did not reach a RER > 1.10. In submaximal exercise, the peakVO_2_ should be interpreted with caution, and the VO_2_ at AT needs to be used to assess the functional capacity and prognosis. Unfortunately, identifying the anaerobic threshold may be associated with substantial error and uncertainty. In this study, the OUES was used as a submaximal and effort-independent parameter as it is easier to determine than the anaerobic threshold and unlike the peakVO_2_ does not rely on the last segment of the exercise. Instead, it uses all of the exercise data, even if they are submaximal, which can be an advantage in patients who do not tolerate maximal exertion, particularly Fontan patients with a worse functional class, PLE, or low motivation to finish the test.

In certain patient groups, however, the OUES values must be interpreted with caution. Firstly, Giardini et al. [39] observed that in cyanotic Fontan patients, the OUES values calculated from the first and last 50% of the entire exercise duration differ substantially. Secondly, the OUES values are considerably influenced by anthropometric variables and show large inter-individual variation. The interpretation of its values is dependent on comparisons with adequate reference values, comparisons between subjects, or comparisons within subjects. It is still unclear which values, namely absolute or indexed to weight, height, age, BSA, or predicted percentage, can best be used [40]. Finally, the OUES and peakVO_2_ are not necessarily interchangeable parameters. However, the OUES is not meant to predict the maximal exercise parameters; it provides an objective and independent measure of the cardiorespiratory function and it seems to be a useful submaximal alternative in Fontan patients unable to perform maximal exercise.

#### 4.1.3. Oxygen Pulse Kinetics

In our series, the patients with Fontan circulation had a lower oxygen pulse, with a mean of 66% of the predicted value, suggesting a stroke volume limitation. Furthermore, in half of the cohort, the rise in the oxygen pulse reached a plateau much earlier at the AT and then flattened.

The oxygen pulse is the product of the stroke volume and arteriovenous oxygen difference during exercise. It represents the oxygen consumption per heartbeat and is considered a surrogate of the stroke volume in the absence of anemia or severe hypoxemia. During the initial and intermediate phases of exercise, the stroke volume has a higher relative contribution to the cardiac output, although at a certain point it stops increasing and reaches a plateau. At low exercise loads, the cardiac output increases due to an increase in stroke volume and HR. At higher loads, the stroke volume no longer increases and the rise in cardiac output is due to an increase in heart rate. Thus, the early flattening or decreasing O_2_ pulse curve in the CPET reflects a cardiac limitation to increase the stroke volume during exercise or a peripheral limitation of the O_2_ extraction in the skeletal muscles. It is noteworthy that the stroke volume also represents the amount of ventricular filling during diastole and may, therefore, be limited by diastolic factors.

In line with the study by Bansal et al. [41], early flattening of the O_2_ pulse slope or a downward displacement at peak exercise in our series was associated with lower O_2_ consumption. This was likely due to the Fontan circulation’s inability to increase the pulmonary blood flow and ventricle preload during exercise, and as a consequence a limited capacity to increase the stroke volume with exertion [42,43,44,45,46]. Furthermore, we observed that those with flat or descending oxygen pulse kinetics had a lower functional capacity. Therefore, we have seen that the morphology of the oxygen pulse curve is a marker of functional capacity in these patients, reflecting a reduced stroke volume reserve.

#### 4.1.4. Heart Rate Response

Chronotropic insufficiency is common in patients with Fontan circulation (up to 85% in our study) and has been associated with exercise limitations [47]. However, the correlation between the HR and functional capacity was modest and the oxygen pulse values were not found to be statistically different between patients with and without chronotropic insufficiency, as in other studies [41,47].

In Fontan patients, it has been shown that the sinus node may be dysfunctional, either congenitally or by damage caused by multiple surgeries (direct damage or indirectly by damaging the arterial supply or innervation). Thus, sinus node dysfunction is common and chronotropic incompetence has been associated with exercise limitations in this patient population. However, since there is no optimal definition for chronotropic incompetence, identifying patients in whom exercise limitations are causally related to chronotropic incompetence and who would, therefore, benefit from pacing remains a challenge. Previous studies have used the term “chronotropic incompetence” when the peak exercise HR failed to reach a lower arbitrary percentage (either 85%, 80%, or less commonly 70%) of the age-predicted maximal HR. Nevertheless, it does not necessarily mean that a reduced peak heart rate during exercise is directly responsible for exercise intolerance. Other parameters such as inadequate effort, residual cardiac shunting, inadequate systemic ventricular filling, or inappropriate ventricular contraction also modulate the exercise capacity and peak heart rate. Consistent with this, in our series the HR reserve (HRR) was lower in Fontan patients as compared with controls, even though up to 92% of the patients did achieve a maximal effort. Thus, Claessen et al. [48] demonstrated that the HRR was impaired in a selected cohort of well-functioning Fontan patients as compared to controls. However, the HR response was appropriate relative to the exercise intensity and higher at any given value of VO_2_ than in the healthy subjects. They also showed that the stroke volume (SV) decreased during exercise in the Fontan patients, whereas the controls showed an increase in SV during exercise. The clinical implications of these findings would be that medications resulting in relative bradycardia during exercise may lead to improved exercise capacity because of enhanced diastolic filling, whereas chronotropic medications or pacing would be expected to have negative effects.

### 4.2. Physical Activity Level

We saw a progressive increase in the peak%VO_2_ predicted as the physical activity level (as determined by the IPAQ questionnaire) increased in both the FPs and HCs. Although there were differences in peakVO_2_ between the three levels of physical activity, a statistically significant difference was only observed between the low and high intensity levels (Figure 1). This could be explained by the small sample size. However, another explanation could be related to the self-reported overestimated physical activity as assessed by the IPAQ as compared to objective measurements in patients with congenital heart disease [49]. Despite this limitation, with a high negative predictive value, the IPAQ is a potentially useful tool for detecting patients with insufficient physical activity level.

Two patients in our cohort showed a peakVO_2_ greater than 80% of the predicted value, which has been dubbed as “super Fontan”. These patients showed a normal O_2_ pulse, normal VO_2_ at the AT, and normal HR response with exertion. Both patients had a dominant left ventricular morphology and a high level of regular physical activity, factors previously associated with increased physical performance and this “super Fontan” phenotype [50]. Regular participation in moderate and high intensity sports is important for the proper development of skeletal muscle mass and prevention of sarcopenia, as the peripheral skeletal muscles act as a pump to boost venous return and increase preload and ventricular filling [51]. A muscle mass deficit also affects the peripheral oxygen extraction.

Although further studies are necessary, exercises that maintain muscle mass, especially lower extremity muscle mass, should be encouraged. Moreover, the recent guidelines from the American Heart Association and the Cardiac Society of Australia and New Zealand emphasize the importance of regular physical activity and exercise in individuals with Fontan circulation [52].

The findings to date have suggested that exercise training can have important benefits for patients with Fontan circulation in both single left and right ventricles [53,54]. Although beyond the scope of this study, other CPET parameters such as the OUES, O_2_ pulse, or HR response might help personalize exercise prescriptions for this patient population. Nevertheless, our study is really only a pilot investigation and further research is required to assess the optimal training type, intensity, duration, and long-term effects.

### 4.3. Lung Mechanics

Restrictive lung disease is common in individuals with a Fontan circulation, and several studies have shown reduced FEV1 and FVC values and a normal or high FEV1/FVC ratio in this patient population [5,6,7,8].

In Fontan patients, lung development may be adversely impacted by a variety of factors commonly seen in patients with congenital heart disease, including oxygen desaturation, mechanical ventilation, lymphatic dysfunction, multiple sternotomies and thoracotomies, scoliosis or pectus deformity, and postoperative complications such as pleural adhesions and diaphragmatic palsy [9].

Overall, the Fontan patients in our series showed smaller lung volumes compared to the healthy controls. Seventy-three percent of the patients presented with a restrictive pattern during spirometry and having a previous lateral thoracotomy was significantly associated with impaired lung function. However, in contrast to prior studies, we did not observe an association between a reduced FVC and lower peakVO_2_. Thus, Matthews et al. [5] observed correlations of 0.442 between the peakVO_2_ and FEV1 (*p* = 0.013) and 0.409 for FVC (*p* = 0.022) and Callegari et al. [8] found that reduced FEV1 was associated with a for the reduced peakVO_2_% predicted (r = 0.43; *p* < 0.0001). Moreover, Alonso-Gonzalez et al. [9] found that moderate to severe impairment of lung function was an independent predictor of survival in a large cohort of adults with congenital heart disease.

In line with this, Turquetto et al. [6] found a strong correlation between lung function and the absolute peakVO_2_ (FVC (r = 0.86, *p* < 0.001); FEV1 (r = 0.83, *p* < 0.001)) in a cohort of Fontan patients, with prevalence rates of moderate restriction of up to 44%. Although an insufficient sample size to demonstrate significant differences may be the cause, since in our series the majority of patients had only a mild restrictive pattern, the low prevalence of moderate restrictive disease (only 16.2%) can also explain this discrepancy.

Having abnormal lung mechanics also impairs the negative intrathoracic pressure required to “pull” blood through the Fontan circulation [12]. In this patient population, in which there is a compromised pulmonary function mainly due to a restrictive pattern, as we confirmed, and a low functional capacity, as indicated by a low peakVO_2_, it appears that the skeletal muscle and ventilatory pumps account importantly for the increase in cardiac output during submaximal exercise in patients with Fontan circulation. It has been previously reported that patients with CHD often have an abnormal body composition [10,11]. Compared with their healthy peers, these individuals have reduced muscle mass, increased adiposity, and a shorter stature. Using the maximal inspiratory pressure (MIP) and sniff nasal inspiratory pressure (SNIP), we found that the inspiratory muscle strength was impaired in Fontan patients compared to healthy controls. However, the role of inspiratory muscle training in the exercise capacity of Fontan patients remains unclear. Although Greutmann et al. [55] found a weak but significant correlation between the MIP and peakVO_2_ (r = 0.33, *p* = 0.03) in a subgroup of 11 Fontan patients, we found no correlation between the MIP and peakVO_2_, nor could it be demonstrated by Turquetto et al. [6]. Further research needs to be done to assess the benefits of respiratory training in exercise performance.

### 4.4. Limitations of the Study

We must acknowledge several limitations. The small sample size of the population study reduces the robustness of the results. However, the sample was homogeneous in terms of age, and despite the small number there was a significant difference between the patients and healthy controls. It is noteworthy that in patients with congenital heart disease, the IPAQ tends to overestimate and physical activity level [49]. Limitations notwithstanding, the IPAQ remains a useful tool to determine the level of physical activity and does not preclude drawing conclusions in this population.

Data on static lung volumes measured by plethysmography were not available for the scope of this study. However, forced expiratory and inspiratory lung flows in ACHD patients were recorded by trained physicians following the American Thoracic Society guidelines. Another potential limitation is the much higher proportion of patients with left single ventricle morphology included in our study. Together with the small sample size, this limits the ability to account for confounding variables such as age, the ejection fraction, or the atrioventricular valvular function. The assessment of cardiac function during exercise may have shown stronger correlations with peakVO_2_. However, our study did not specifically address the ventricular function or hemodynamic abnormalities and their potential contributions to exercise intolerance. Finally, a follow-up study may allow for us to identify the prognostic value of the CPET parameters and inspiratory muscle function in the outcome of Fontan patients.

## 5. Conclusions

Patients with Fontan circulation have an impaired exercise capacity. The OUES is a useful parameter of functional capacity in both patients who fail or not to reach maximal exertion, showing a strong positive correlation with the peakVO_2_. Chronotropic insufficiency and an early flattened or descending oxygen pulse slope is associated with lower peakVO_2_, whereas left ventricular morphology associates with better functional capacity. Those patients who perform regular intense physical activity are associated higher exercise capacity, highlighting the importance of regular exercise training. Although patients with Fontan circulation have inspiratory muscle weakness, its impact on their functional capacity is unclear.

## Figures and Tables

**Table 1:** The Fontan patients’ baseline characteristics.

	Total (*n* = 37)
**Mean age**	26.5 ± 6.25
**Sex**	
**Men**	25 (68%)
**Women**	12 (32%)
**Smoking**	3 (8%)
**Pectum excavatum**	3 (8%)
**Ventricular type**	
**LV**	30 (81%)
**RV**	5 (13.5%)
**Balanced**	2 (5.5%)
**Pulmonary Banding**	8 (21.6%)
**SP shunt**	17 (46%)
**Lateral Thoracotomy**	24 (65%)
**Fontan type**	
**Intracardiac**	4 (11%)
**Extracardiac**	30 (81%)
**Lateral tunnel**	3 (8%)
**Fenestration**	13 (35%)
**Age at Fontan**	8.2 (6.1–11)
**Years after Fontan**	17 ± 5.7
**Pacemaker**	4 (10.8%)
**Drugs**	
**Beta-blocker**	7 (19%)
**ACEi/ARBs**	5 (13.5%)
**Aldosterone antagonist**	5 (13.5%)
**Diuretics**	4 (11%)
**Antiarrhythmic**	0
**PDE5i**	3 (8%)
**ERA**	2 (5.4%)
**Antiplatelet**	34 (92%)
**Oral anticoagulant**	4 (11%)

Data are shown as means ± standard deviations, medians (Q1–Q3), or percentages. Note: *p*-value: Student’s *t* or Mann–Whitney U test. ARB: angiotensin receptors blockers; ACEi: angiotensin-converting enzyme inhibitors; ERA: endothelin receptor antagonist; LV: left ventricle; PDE5i: phosphodiesterase 5 inhibitors; RV: right ventricle; SP shunt: systemic to pulmonary shunt.

**Table 2:** Lung function and inspiratory muscle strength results.

	Fontan Patients (*n* = 37)	Healthy Subjects (*n* = 19)	*p*
**FEV1 (%)**	78.8 ± 12.4	95.6 ± 6.5	<0.001
**FVC (%)**	75 ± 11.3	91 ± 9.5	<0.001
**FEV1/FVC**	88 ± 6.4	87.3 ± 8	0.748
**MIP (cmH_2_O)**	79 (66–97)	102 (84–125)	0.005
**MIP (%)**	78 (65–91)	97 (84–128)	0.006
**SNIP (cmH_2_O)**	74 (60–88)	89 (81–107)	0.004
**SNIP (%)**	77 (59–88)	94 (88–102)	0.001

**Table 3:** Cardiopulmonary exercise test parameters and IPAQ results.

	Fontan Patients (*n* = 37)	Healthy Subjects (*n* = 19)	*p*
**Age**	26.5 ± 6.25	26.1 ± 6.8	0.808
**Weight**	67 ± 13	66 ± 13	0.753
**Height**	1.69 ± 0.06	1.72 ± 0.08	0.226
**BMI (kg/m^2^)**	23 ± 4	22 ± 3	0.257
**PAL (IPAQ)**			0.461
**Low**	10 (27%)	7 (37%)	
**Moderate**	22 (59.5%)	8 (42%)	
**Intense**	5 (13.5%)	4 (21%)	
**pStO_2_ basal**	97 (95–99)	100 (99–100)	<0.001
**pStO_2_ max**	91 (89–93)	98 (97–99)	<0.001
**Peak load (W)**	128 ± 29	209 ± 49	<0.001
**Peak load (%)**	73 ± 18	115 ± 17	<0.001
**RER max**	1.18 ± 0.08	1.22 ± 0.07	0.129
**PeakVO_2_ (mL/kg/min)**	21 ± 5.4	34.7 ± 6.1	<0.001
**PeakVO_2_ (mL)**	1406 ± 330	2363 ± 635	<0.001
**PeakVO_2_ (%)**	55.5 ± 14	87.6 ± 11	<0.001
**VO_2_ AT (mL/kg/min)**	12.5 ±3.2	19.8 ± 4.2	<0.001
**VO_2_ AT (%)**	32.3 ± 8	50 ± 8	<0.001
**VO_2_/W slope**	8.8 ± 1.2	10 ± 1.2	0.001
**OUES (** **mL/min/log(L/min))**	1608 ± 359	2594 ± 700	<0.001
**OUES (%)**	54.6 ± 13	83 ± 15	<0.001
**OUES/kg**	23 (20–28)	41 (32–43)	<0.001
**Peak HR**	157 ± 16	180 ± 16	<0.001
**MPHR (%)**	82 ± 9.3	94 ± 6.7	<0.001
**Chronotropic index**	0.65 ± 0.15	0.89 ± 0.12	<0.001
**VE max**	60.4 ± 12.6	91 ± 25	<0.001
**Peak Bf**	36 ± 8	41 ± 10	0.088
**Vt max**	1.78 ± 0.44	2.32 ± 0.66	<0.001
**Vt/VC (%)**	49.8 ± 10.7	50 ± 7	0.871
**Breathing Reserve**	50 ± 12.7	33 ± 14	<0.001
**MVV**	125 ± 27.4	156 ± 28	<0.001
**VE/VCO_2_ AT**	30.4 ± 3.5	24.7 ± 2.3	<0.001
**PETCO_2_ AT**	36 ± 4	44 ± 5	<0.001
**VE/VCO_2_ slope**	28 ± 4.5	23.5 ± 3.2	<0.001
**Oxygen debt**	46.2 ± 9.3	36 ± 5.4	<0.001
**VO_2_RD**	10 (5–15)	5 (5–10)	0.024
**HR recovery**	22 ± 9.8	22 ± 9	0.870
**Oxygen pulse (%)**	65.7 ± 13.8	92 ± 14	<0.001
**Oxygen pulse (mL/beat)**	8.7 ± 1.8	13 ± 3.4	<0.001

Data are shown as means ± standard deviations, medians (Q1–Q3), or percentages. Note: *p*-values: Student’s *t* or Mann–Whitney U test. AT: anaerobic threshold; BMI: body mass index; Bf: breathing rate; HR: heart rate; kg: kilograms; L: liters; log: logarithm; m^2^: square meters; ml: milliliters; min: minute; MPHR: maximal predicted heart rate; MVV: maximal ventilatory volume; OUES: oxygen uptake efficiency slope; PAL: physical activity level; PETCO_2_: CO_2_ end-expiratory pressure; RER: respiratory exchange ratio; VC: vital capacity; VE: minute ventilation; VCO_2_: CO_2_ production; VO_2_: oxygen uptake; VO_2_RD: VO_2_ recovery delay; Vt: tidal volume; W: watts.

## Data Availability

The data underlying this article cannot be shared publicly due to data privacy reasons and the according regulations.
